# Genetic Elucidation of Ultrasonography Fetal Anomalies in Children with Autism Spectrum Disorder

**DOI:** 10.1192/j.eurpsy.2023.282

**Published:** 2023-07-19

**Authors:** O. Regev, A. Shil, T. Bronshtein, A. Hadar, G. Meiri, H. Flusser, A. Michaelovski, I. Dinshtein, R. Hershkovitz, I. Menashe

**Affiliations:** 1 Ben-Gurion University; 2 Clalit Health Services; 3 Soroka University Medical Center, Beer Sheva, Israel

## Abstract

**Introduction:**

Autism spectrum disorder (ASD) is a highly heritable neurodevelopmental disorder affecting 1-2% of the population worldwide. Recent large-scale whole-exome sequencing (WES) studies identified hundreds of rare, highly penetrant genetic variations associated with ASD. Many of these genetic variations underlie particular genetic syndromes characterized by a variety of congenital anomalies in addition to the core ASD symptoms. Recently, we reported about certain ultrasonography fetal anomalies (UFAs) associated with later development of ASD (Regev *et al.* Brain 2022).

**Objectives:**

To identify genetic mutations associated with UFAs in children with ASD.

**Methods:**

We conducted a cross-sectional study of all children diagnosed with ASD registered at the Azrieli National Centre for Autism and Neurodevelopment (ANCAN) who have both fetal ultrasound and WES data. We used an integrative in-house bioinformatics pipeline specifically designated to identify gene-disrupting variants (GDVs) in a panel of >1200 genes associated with ASD according to SFARI gene database. Then, we compared the prevalence of GDVs in these genes between children with and without UFAs. Finally, we applied the Gene Analytics tool to disrupted genes in children with specific fetal anomalies to identify biological pathways associated with both ASD and these fetal anomalies.

**Results:**

Overall, 115 ASD children were included in this study, of which 49 (42.6%) of them had UFAs in their ultrasound scans (Figure 1). Children with and without UFAs did not differ in their sociodemographic and clinical characteristics except for a significantly lower proportion of males in the UFA group (63.4% vs. 84.8%, respectively; p=0.011). Notably, **children with UFAs were more likely to carry GDVs in ASD genes than their counterparts** even after adjustment to the sex differences between the groups (aOR=2.27, 95%CI: 1.05-4.93), and this association was the most prominent with GDVs in the most notable ASD genes (i.e., those with SFARI gene score=1). Also, the study shows **higher prevalence of children with GDVs in most anatomical systems, with UFAs in fetal size (14.8% vs. 1.6%, p=0.012, cases vs. controls) and the head&brain (16.7% vs. 4.9%, p=0.040, cases vs. controls)** being the most prominent (Figure 2). In addition, **children with UFAs had significantly more co-occurring mutations, and the number of mutations in a single fetus was significantly correlated with the number of UFAs (r=0.20, p=0.035)**.

**Image:**

**Figure:**
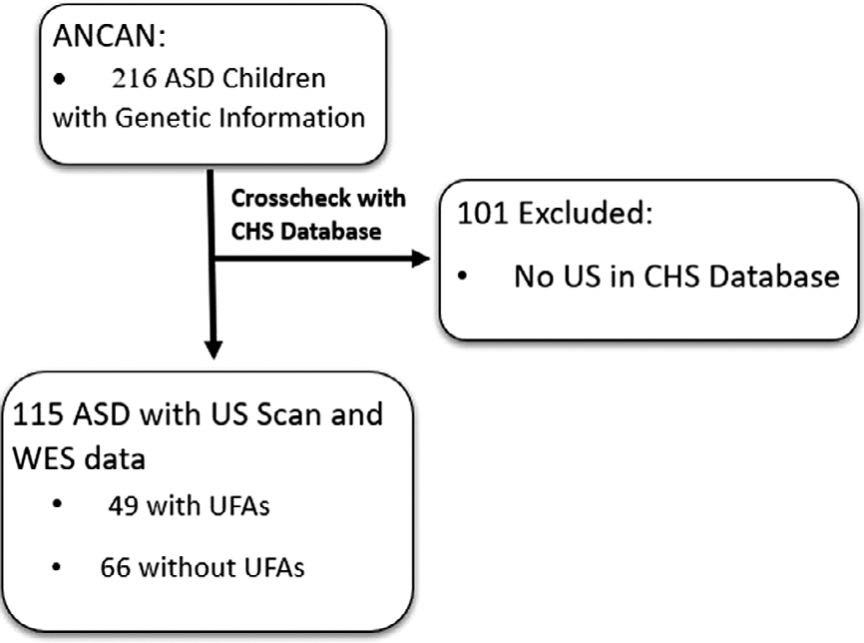

**Image 2:**

**Figure:**
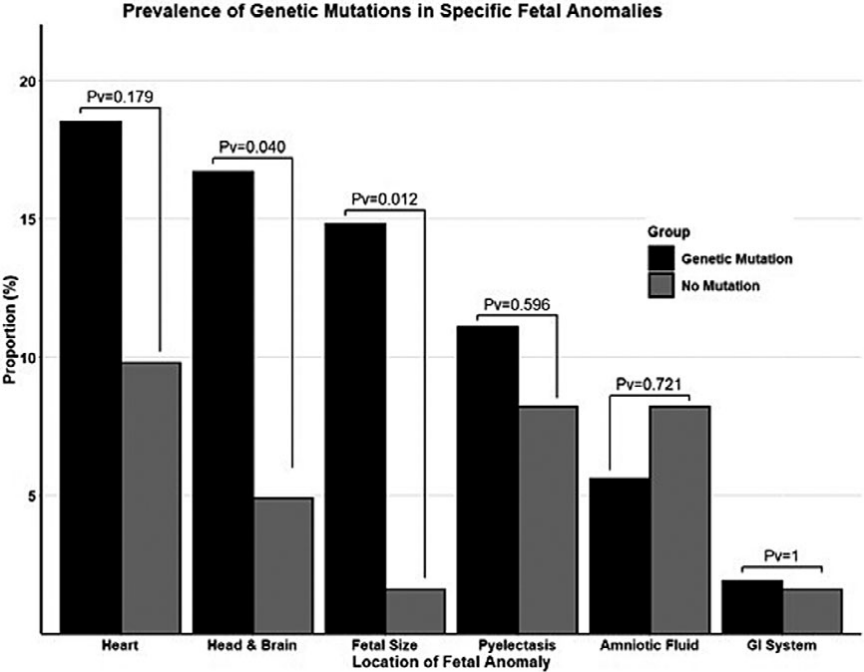

**Conclusions:**

Our findings suggest distinct genetic mechanisms for ASD subtypes that are characterized by unique UFAs. These findings may form a basis for future prenatal screening approaches for ASD using both ultrasound and genetic testing. Our findings suggest distinct genetic mechanisms for ASD subtypes that arecharacterized by unique UFAs. These findings may form a basis for future prenatal screening approaches for ASD using both ultrasound and genetic testing.

**Disclosure of Interest:**

None Declared

